# Surface Stickiness Perception by Auditory, Tactile, and Visual Cues

**DOI:** 10.3389/fpsyg.2019.02135

**Published:** 2019-09-18

**Authors:** Hyungeol Lee, Eunsil Lee, Jiye Jung, Junsuk Kim

**Affiliations:** ^1^Department of Philosophy, Sogang University, Seoul, South Korea; ^2^Department of Psychology, Duksung Women’s University, Seoul, South Korea; ^3^Department of Psychology, Chung-Ang University, Seoul, South Korea; ^4^Center for Neuroscience Imaging Research, Institute for Basic Science, Suwon, South Korea; ^5^Department of Biomedical Engineering, Sungkyunkwan University, Suwon, South Korea

**Keywords:** texture perception, surface stickiness, auditory cues, tactile cues, visual cues

## Abstract

This study aimed to explore the psychophysical bases of multisensory surface stickiness perception by investigating how sensitively humans perceive different levels of stickiness intensity conveyed by auditory, tactile, and visual cues. First, we sorted five different sticky stimuli by perceived intensity in ascending order for each modality separately and evaluated the discrimination sensitivities of each participant using a fitted psychometric curve. Results showed that perceptual intensity orders were not identical to physical intensity order and that the sequential order of perceived intensities for different modalities was inconsistent. Moreover, estimated perceptual sensitivities to surface stickiness indicated that auditory cues result in better discrimination sensitivity than tactile and visual cues. Second, we calculated the relative perceptual distances of stickiness intensities using multidimensional scaling. A follow-up statistical test demonstrated that the perceptual mapping of vision and touch are similar but that auditory perception is different. These results suggest that the discriminability of stickiness intensity is best served by auditory cues and that texture information processing in the auditory domain is distinctive from that of other modalities.

## Introduction

To interact effectively with surrounding objects, humans need to acquire surface texture information from objects using different sensory modalities. For example, when perceiving surface characteristics of a certain object, we could obtain texture information (1) by looking at the surface, (2) by touching it with our hands, or (3) by hearing sounds generated by interacting with it. Previous research has shown that information from the visual channel, relative to other modalities, is weighed most strongly for object perception (Heller, 1982) and this also applies to surface texture perception (Tiest and Kappers, 2007). On the other hand, it has been demonstrated that tactile information is essential in perceiving the characteristics of surface texture (Lederman and Klatzky, 2009) and auditory cues play important roles in texture discrimination tasks (Lederman and Abbott, 1981; Lederman et al., 2002; Drewing et al., 2004). Based on these previous studies, there appears to be no fixed sensory dominance for texture perception and it seems that sensory dominance is largely dependent on specific aspects of surface texture, e.g., particle size of rough surfaces (Lederman and Klatzky, 2004).

It is well-known that the perception of surface texture is multidimensional, e.g., roughness/smoothness, hardness/softness, stickiness/slipperiness, and warmth/coolness (Hollins et al., 1993; Bensmaia and Hollins, 2005; Bensmaia, 2016). Compared to other dimensions, stickiness is one of the least investigated properties of surface perception (Bensmaia, 2016). Stickiness can be defined as a mechanical sensation related to the friction between skin and surface, or stretch of skin (Bensmaia, 2016). More specifically, it can be sub-divided into non-slipperiness arising from horizontal movement and stickiness stimulated by vertical movement. Note that the current study focuses on stickiness perception evoked by vertical pull-off movements. There are several studies on stickiness perception and its neural mechanism (Zigler, 1923; Kim et al., 2017; Yeon et al., 2017), but they examined solely stickiness from the tactile sense, and *not* from visual and auditory senses. Moreover, in these studies, the authors have used sticky stimuli such as liquid glue, prunes, molasses, and jelly (Zigler, 1923), or silicone-based sticky objects into which fast catalysts were mixed in several proportions (Yeon et al., 2017). All these sticky stimuli are difficult to quantify in terms of the physical intensities of stickiness. To make up for these shortcomings and improve the reproducibility of the research, we used adhesive tapes which are relatively easy to obtain and have stickiness intensity of several levels.

Since this is the first study of the stickiness dimension using various sensory modalities, we formulated a hypothesis based on previous findings of multisensory roughness perception (Lederman and Klatzky, 2004; Klatzky and Lederman, 2010). If the perceptual characteristics of surface roughness hold for stickiness stimuli, we can assume that sensitivity in stickiness perception would be similar for vision and touch and less for the auditory modality (Lederman and Klatzky, 2004). However, there is an obvious difference in visual perception for rough compared to that for sticky surfaces. Roughness intensity can be perceived visually by looking at the surface texture (e.g., particle size or inter-particle distance), whereas it is difficult to measure stickiness intensity by observing the surface texture. Moreover, in our daily life, we are far more familiar with tactile stickiness than with visual and auditory cues for stickiness. Therefore, we hypothesized that the sense of touch would show the highest sensitivity in surface stickiness perception.

In this psychophysical study, we investigated how differently stickiness perception is mediated by auditory, tactile, and visual cues. To be specific, we examined (1) in which modality people are the most sensitive in terms of stickiness intensity discrimination using the just noticeable difference (JND), (2) the relative similarity of stickiness intensities across modalities using multidimensional scaling (MDS), and (3) the relationship between physical and perceived stickiness intensities.

## Materials and Methods

### Participants and Ethics Approval

Twenty-seven volunteers (10 females, 24.52 ± 2.79 years old, age range: 20–34 years) participated in this experiment. All participants were all right-handed and had no deficits in auditory, tactile, and visual processing. Experimental procedures were approved by the Ethical Committee of Sungkyunkwan University (IRB# 2018-05-001) and the study was conducted in accordance with the Declaration of Helsinki. All participants were informed about the experimental procedure and gave written informed consent prior to their participation.

### Stimuli

Five different kinds of repositionable tape (9415PC, 665, 9183, 9495, and 9071; 3M Center, St. Paul, MN, United States) were prepared for the experiment. To measure physical stickiness strength of these tapes, we employed a “probe tack test” that measures the peak value of adhesive force indicating the instantaneous adhesion property. Using a probe in the shape of a stainless steel ball (1-inch diameter), we measured the adhesive force that occurs when a probe is peeled off at peeling rate 2 mm/sec. Previous studies have considered this test as a qualitative approach to evaluate tactile sensations of human (Mith et al., 2008; Cakmak et al., 2011). The physical stickiness intensities of tape 9415PC, 665, 9183, 9495, and 9071 were estimated as 22.9, 124.5, 330.0, 419.2, and 558.7 gf (gram-force), respectively. According to these values, we labeled five different physical stickiness intensities as level 1 to level 5: 9415PC (level 1), 665 (level 2), 9183 (level 3), 9495 (level 4), and 9071 (level 5). With these tapes, we created auditory, tactile, and visual stimuli.

**FIGURE 1:**
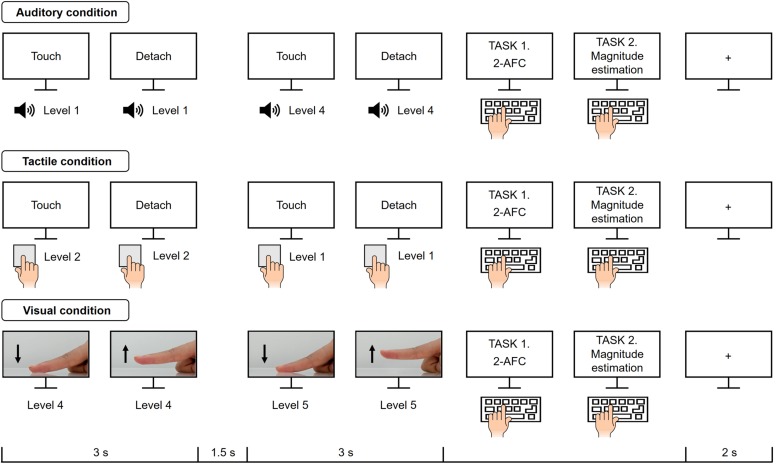
Structure and time course of the experimental design. Participants completed five blocks per modality condition. Following the instruction on the screen, participants perceived stickiness intensity levels through the sensory cues, i.e., auditory, tactile, and visual cues. In each trial, two stimuli were presented one after another for 3 s each and then participants were asked to respond to questions (1) “Does the second stimulus feel stickier than the first one?” and (2) “How much stickier was the second stimulus compared to the first one?”.

• Auditory stimuli: Sound clips of tactile explorations (i.e., a sound when touching and detaching with an index finger) were recorded with a mobile condenser microphone. Each clip lasted 3 s and was sub-divided into a touching and then a detaching period of about 1.5 s each.
• Tactile stimuli: Tapes were prepared in a size of 5 × 1.9 cm and attached to an acrylic plate sized 5 × 8 cm. The plastic plate was used to enable the experimenter to present the stimuli easily and without direct contact. All tactile stimuli were used only once, then replaced by new tape.
• Visual stimuli: Video clips of tactile explorations were recorded with a resolution of 1920 × 1080 at 60 frames per second (Figure 1). Each clip displayed a right index finger touching one of the five tapes and lifting off. The recording video camera was positioned at a distance of 20 cm from the stimulus surface and 5 cm above the tabletop. Each video clip lasted 3 s.

Note that we did not match the absolute perceptual intensities across sensory modalities. We were aware of that stimulus intensities can be perceived in a different scale depending on the modalities, but the primary consideration was that our stimuli should be expressed its unique characteristics as fully as possible.

### Experimental Design

Twenty-five paired stimuli (counterbalanced pairwise combinations of five distinct intensities, plus five pairs of the same intensity; _5_P_2_ + 5 = 25) were used for each modality condition and these pairs were presented once in each block. Participants performed 5 blocks for each modality, thus there were a total of 375 trials (3 modalities × 5 blocks × 25 pairs). A single trial presented two stimulus pairs one after another for 3 s and there was a pause of 1.5 s between them (Figure 1). Participants were instructed to perceive stickiness intensity from the sensory cues (auditory, tactile, and visual information) and to conduct two different tasks, i.e., 2-Alternative Forced Choice (2-AFC) and magnitude estimation of perceived dissimilarity. The 2-AFC and the magnitude estimation are the common psychophysical methods for measuring the subjective experience (Lederman and Klatzky, 2009). In tactile conditions, participants performed tasks following the instruction “Touch” and “Detach” displayed on a monitor. To give more detail, a tactile stimulus was given to the participants prior to the stimulation periods. When “Touch” was on the monitor, participants put their index finger on the tape and lifted off as soon as they saw “Detach” on the monitor.

• 2-AFC: Participants were asked to respond to the question “Does the second stimulus feel stickier than the first one?” If the second stimulus was felt to be stickier, participants pressed a “Yes” button on the keyboard, otherwise they pressed a “No” button.
• Magnitude estimation: Participants were asked to respond to the question “How much stickier was the second stimulus compared to the first one?” The response range was from −10 to 10. Participants reported a negative value if the second stimulus was felt to be less sticky and a positive value if the second stimulus was felt to be more sticky. They also could respond 0 if the pair of stimuli were felt to be of the same intensity. The larger the response value, the greater the difference in perceived intensity between the stimulus pair.

A short break was provided between the blocks and the entire experiment took approximately 60 min. The presentation order of stimuli as well as modality was randomized to remove ordering effects. Note that we did not notify participants of the number of intensity levels throughout the entire experiment (Supplementary Material).

### Data Analyses

#### 2-Alternative Forced Choice

We first computed the sequential order of perceived intensity for the five sticky stimuli to find a relationship between stimulus intensity and sensation. In addition, to fit a psychometric curve to the behavioral responses, we calculated “Yes” answer rates for each of the stimulus pairs.

• Ordering perceived intensity: To test whether the order of perceived intensity is consistent with the physical intensity order, we compared the average number of “Yes” responses for each stimulus pair. Our experiment asked the participants to answer whether the secondly presented stimulus was felt stickier than the first one. To minimize these sequential effects of stimulus presentation, we presented each pair of stimuli in both ascending and descending order, e.g., level 3 - 4 and level 4 - 3. For example, if the average number of “Yes” across participants was 4.13 for a stimulus pair of levels 3 - 4 and 2.58 for a pair of level 4 - 3, we considered that the perceived intensity of level 4 was greater than the perceived intensity of level 3. Through this process of comparing average number of “Yes” answer for every stimulus pair, the ascending order of perceived intensity of stimuli was obtained.
• “Yes” answer rates: These rates were derived by the ratio of the number of “Yes” responses to the number of stimulus pairs presented. For example, if the “Yes” answer rate was 0.3, it meant that the second stimulus was perceived to be stickier three times when a stimulus pair is presented ten times.

Psychometric curves were fitted to the “Yes” answer rates. In particular, to set the values of the *x*-axis, we assumed that the intensity difference between perceptually adjacent stimuli has a quantity of 1 regardless of the actual physical intensity difference. Hence, the *x*-axis was set to indicate the perceived intensity difference ranging from −4 to 4, and the y-axis indicated the “Yes” answer rates. A psychometric function was derived using the Palamedes toolbox for Matlab, which implements a maximum-likelihood method (Wichmann and Hill, 2001; Prins and Kingdom, 2018). It showed a cumulative probability that the stickiness intensity of the second stimulus was perceived to be stickier than the first stimulus, as a function of its relative stickiness. Therefore, a steeper slope indicated a better discriminability. Specifically, the discriminability of each participant was estimated as a difference of perceived intensity values between the 25th and 75th percentiles. This difference is known as the just noticeable difference, i.e., JND. To determine the reliability of the JND, we estimated each participant’s goodness of fit. We fitted a logistic function to the psychometric curve and the individual deviances were evaluated to measure goodness of fit (Fruend et al., 2011). A 95% confidence interval was calculated based on simulations from the bootstrapping procedure (*n* = 1000). If the observed deviance was outside the 95% confidence limit, we considered the participant’s data as an outlier.

#### Magnitude Estimation

To compute a dissimilarity matrix for each modality, we normalized participants’ responses in the magnitude estimation tasks so that the mean value became 0 and the standard deviation became 1. The normalized responses were averaged across participants to obtain a group-level dissimilarity matrix. An entry at the *j*-th row and the *k*-th column of the dissimilarity matrix was the average perceived difference in a normalized scale when the *j*-th intensity was presented followed by the *k*-th intensity. Then, an MDS was applied to this dissimilarity matrix to yield the spatial organization of perceptual responses for the five different stickiness intensities.

To further examine the correspondence of perceived dissimilarities between sensory modalities, we employed the Mantel test to calculate a correlation between the matrices (Saarimaki et al., 2016). The significance was tested from the probability distribution obtained from 10,000 repeated permutations. For each permutation, we permuted the values of the dissimilarity matrix and determined the expected distribution of the statistics under the null hypothesis. The probability of the observed correlation arising by chance was then yielded by observing where the statistic calculated from our data fell in the permuted distribution.

## Results

### 2-Alternative Forced Choice

**FIGURE 2:**
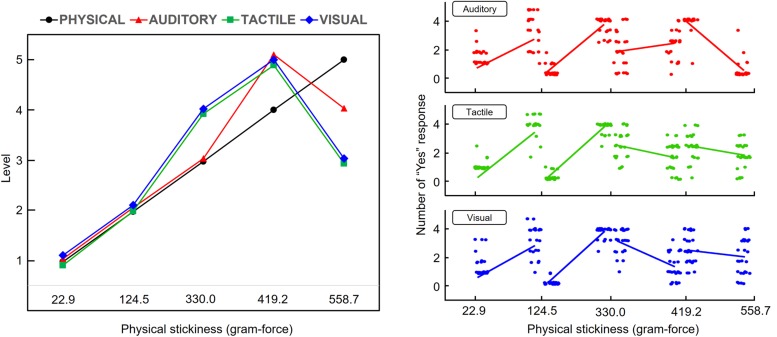
Sequential order of physical and perceived intensities for the 5 sticky tapes. **The left side:** Based on participants’ responses to the 2-AFC task, the 5 stickiness intensities were sorted in ascending order for each modality condition. **The right side:** The number of “Yes” responses of 27 participants are depicted over the physical stickiness values. Each dot indicates a participant. The solid lines indicate the average number of “Yes” responses of each stimulus pair for both ascending and descending order.

Figure 2 shows the sequential order of perceived intensity for 5 sticky tapes and the number of “Yes” responses of each participant (Weissgerber et al., 2015). It is noticeable that physical and perceived intensities did not coincide. More interestingly, the perceptual intensity orders of the three sensory modalities were not identical, i.e., the order of perceived intensity levels for auditory perception differs from those for visual and tactile perception. Based on the ascending order of physical intensity levels (i.e., 1-2-3-4-5), the order for auditory perception was shown to be 1-2-3-5-4, and for tactile and the visual perception was shown to be 1-2-5-3-4.

The JNDs, the point of subject equality (PSE), and the goodness of fit values (i.e., deviance) of all participants across modalities are summarized in Table 1. Among the data of the 27 participants, data from participants 15 and 18 were not well-fitted and we therefore excluded them from the analyses. The mean and the standard error values in the table were calculated after excluding the outlier data. We carried out a one-way repeated measures ANOVA on JND values and found a significant difference in the values for each modality (*F*_2__,__75_ = 20.1, *p* < 0.01). Tukey’s *post hoc* test revealed that JND values for the auditory condition were significantly smaller than those for the visual and tactile conditions (*p* < 0.01). No significant difference was found between visual and tactile conditions.

**TABLE 1:** A summary of the behavioral responses (2-AFC task).

	**Auditory**			**Tactile**			**Visual**		
			
**Participant**	**JND**	**PSE**	**Deviance**	**JND**	**PSE**	**Deviance**	**JND**	**PSE**	**Deviance**
1	0.55	0.43	0.21	2.15	0.74	11.06	2.59	–0.03	6.40
2	1.39	0.12	6.81	2.65	0.21	3.54	3.04	–0.22	6.66
3	1.43	–0.26	10.04	2.11	0.35	3.64	2.86	–0.69	3.72
4	0.97	0.71	5.86	2.08	0.81	12.26	1.46	0.12	3.42
5	1.68	0.84	7.47	1.35	1.39	2.50	2.37	1.63	6.12
6	0.84	0.46	6.03	1.77	0.76	5.91	2.44	–0.14	4.89
7	1.42	–0.31	2.51	1.94	–0.45	3.60	2.48	–0.72	6.09
8	1.40	0.36	4.69	2.23	–0.08	6.28	1.84	–0.33	9.50
9	1.23	–0.21	10.38	2.09	0.13	3.14	2.27	–0.53	13.64
10	1.43	1.12	13.19	3.58	1.01	10.03	2.15	–1.20	5.92
11	1.50	0.27	12.21	2.30	0.03	9.13	2.57	–0.26	7.80
12	2.78	1.34	6.17	2.67	0.03	9.43	2.27	0.30	4.26
13	1.04	0.29	1.71	1.67	0.17	1.93	2.20	0.52	6.36
14	1.37	0.21	6.19	2.67	–0.03	4.93	1.54	0.07	0.91
15^∗^	1.39	–0.12	16.66^∗^	3.08	0.67	20.53^∗^	2.16	0.03	6.30
16	0.85	0.79	0.71	2.45	–0.43	7.73	3.12	–0.29	3.28
17	1.66	0.68	3.41	2.09	–0.03	8.61	1.68	–0.03	5.11
18^∗^	1.84	0.33	16.27^∗^	2.48	0.32	5.46	2.59	0.15	6.20
19	1.66	0.68	3.41	2.22	–0.05	4.45	2.17	0.41	4.03
20	1.19	0.30	3.22	2.12	–0.25	6.76	2.00	0.24	5.62
21	1.22	0.64	3.92	2.53	–0.43	2.72	2.23	–0.14	2.71
22	1.09	0.07	1.69	1.88	–0.13	2.29	3.45	–0.37	11.71
23	0.81	0.37	5.40	1.87	0.44	10.02	2.51	0.67	15.00
24	1.38	0.17	1.83	1.64	–0.27	2.37	3.13	0.55	1.99
25	2.88	0.21	4.78	2.12	0.79	8.03	1.99	0.94	8.16
26	0.84	0.33	0.73	0.82	0.74	5.35	1.78	0.49	2.68
27	2.59	–0.03	7.79	1.74	–0.18	5.19	2.24	–0.41	5.28
Mean	1.41	0.38	5.22	2.11	0.21	6.04	2.33	0.02	6.05
Stdandard Error	0.59	0.40	3.54	0.52	0.50	3.10	0.51	0.60	3.45

*^∗^Data from participants 15 and 18 were considered as outliers and excluded from the analyses.*

### Magnitude Estimation

**FIGURE 3:**
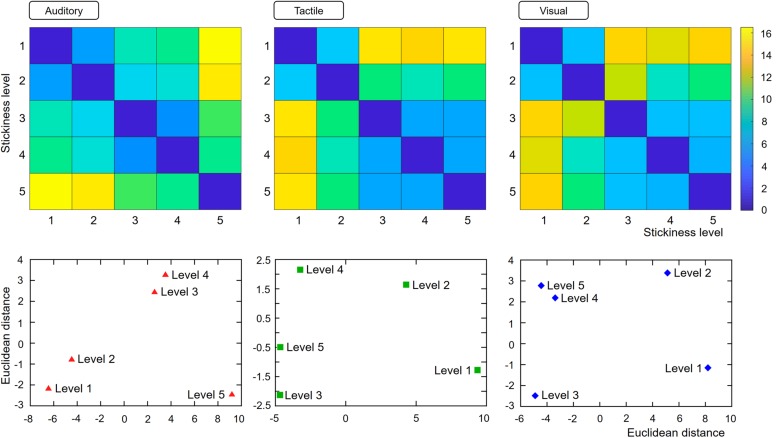
Results of the magnitude estimation task. Distance matrices for each modality condition were computed based on the perceived difference of stickiness intensities. The values in each cell indicate the perceptual distances of each stimulus pair. Based on these values, relative positions of the five stickiness intensities were computed and depicted in a 2-dimensional MDS map.

We computed dissimilarity matrices using perceived intensity differences between stimulus pairs and the corresponding MDS map (Figure 3). To statistically estimate how similarly the spatial configurations were mapped across sensory conditions, we employed the Mantel test to calculate the correlation between modalities. We observed a significant correlation between visual and tactile modalities (*r* = 0.98, *p* < 0.01). However, no other condition-pairs showed any significant correlation (auditory-tactile: *r* = 0.50, *p* = 0.16; auditory-visual: *r* = 0.49, *p* = 0.14).

## Discussion

In this study, we investigated how humans perceive intensity information regarding surface stickiness when it is conveyed via auditory, tactile, and visual cues. Results of the 2-AFC tasks indicated that perceptual stickiness intensities were distinct from physical stickiness intensities. The sequential orders of the perceived intensity for three different modalities were also not identical, suggesting a distinct perceptual process for surface stickiness across sensory modalities. Moreover, we derived JNDs from the psychometric functions for evaluating each participant’s perceptual sensitivity to tactile stickiness. The results indicate that auditory cues resulted in better discriminative sensitivity than tactile and visual cues. Intriguingly, this result contradicts our hypothesis that tactile cues support better perceptual sensitivity. Furthermore, to explore spatial configurations of five levels of stickiness intensity, MDS was applied on the responses in the magnitude estimation tasks. A follow-up Mantel test revealed that the perceptual mapping for vision and touch were statistically similar, but that auditory perception was different. These results thus suggest that the discriminability of stickiness intensity is best served by auditory cues and that texture information processing in the auditory domain is distinctive from that of other modalities.

Our results clearly showed a discrepancy between perceptual and physical stickiness intensities. On the one hand, this may be attributed to the material difference of contacting areas. Participants perceived stickiness intensities by touching with their own fingers or watching/hearing touch by another’s finger. However, for the measurement of physical stickiness, the probe tack test measured the intensity when lifting a steel ball off the surface. Namely, the difference between the physical properties of steel and skin could have brought about dissimilar interactions with the sticky surface, resulting in this discrepancy. On the other hand, the non-linear nature of human intensity perception could cause confusion in the sequential ordering. We observed that the orders of perceived and physical intensity were identical for relatively low physical stickiness intensities (levels 1 and 2) across the modalities, but that this was different for relatively high intensities (levels 3, 4, and 5). In line with the Fechner’s law describing that the magnitude of a subjective sensation increases in proportion with the logarithm of stimulus intensity (Weber, 1996), this observation can be interpreted as participants requiring a greater perceptual difference to discriminate as physical intensity increased. Further studies will be needed to identify various causes that affect surface stickiness perception.

Another key finding of the current study is that participants were most sensitive in perceiving and distinguishing stickiness intensity using auditory sensation. In line with our findings, previous studies have reported that auditory sense plays important roles in texture perception (Bresciani et al., 2005; Avanzini and Crosato, 2006). For instance, Avanzini and colleagues presented sound clips of a contact between a rigid probe and objects with different levels of stiffness and reported that participants’ perceived stiffness was correlated with the auditory information (Avanzini and Crosato, 2006). Moreover, Giordano investigated auditory perception of hardness and demonstrated that contact time is a crucial factor for auditory perception, suggesting that tactile discriminability is strongly dependent on a certain feature of auditory stimuli (Giordano, 2005). Following this, in our experimental paradigm, which feature of the auditory stimuli was beneficial to discriminative performance? There are at least two possible candidates. First, perceived stickiness intensity may be determined by the loudness of stimuli. We examined the physical characteristics of the auditory stimuli and observed a clear interrelationship between amplitude (i.e., loudness) and intensity perception. The amplitudes of auditory stimuli were 57.5, 61.1, 64.3, 71.9, and 64.5 (dB) for levels 1 to 5, respectively. Interestingly, the ascending order of the amplitude values was in accordance with the order of perceived intensities, i.e., 1-2-3-5-4. Hence, it seems that participants perceived intensity according to the loudness of the stimuli. In the case of visual perception, there were far more factors to consider so as to distinguish the intensities, e.g., velocity of vertical movement of the finger and stretching of skin at the moment of lifting off, etc. In the case of tactile perception, factors such as the pressure applied to the sticky surface, or subtle changes in the stickiness as time passes by, might have affected intensity discrimination. Therefore, it is likely that more complex perceptual processes were required for vision and tactile sensation than for auditory sensation. Second, the distinctive perceptual relationship between the five intensities in the auditory domain might lead to a higher discriminability. We investigated the relative positioning of stimulus intensities using MDS and the follow-up Mantel test revealed that the spatial configuration in the auditory domain was different from that of the others. This suggests that the human auditory system has different perceptual processes to that of visual and tactile system, and that auditory information processing could be more efficient for stickiness intensity perception.

Although we tried to minimize potential influences due to our stimuli throughout the experiment, there is still a chance of unexpected confounding. First, we checked the sound onsets carefully and found that there was a subtle difference: The largest timing difference between onsets was 0.003 s. It is unlikely that participants could recognize the timing difference, but we cannot completely rule out its potential influences. Second, the sound of touching a sticky surface was rather loud (e.g., 71.9 dB) considering that the loudness of a normal conversation is approximately 60 dB. Since we focused to capture the clear sound of moment of detachment, we did not notice the loudness of the sound. Third, the sensitivity of tactile stimuli may be varying depending on the size of the tape or the area of the finger. Prior to the experiment, participants practiced finger postures following instruction of the experimenter to standardize their finger movements across participants as well as across trials. However, we cannot completely rule out the unexpected confounding due to the stimuli and movements.

In this study, we investigated how cues in different sensory modalities (auditory, tactile, and visual cues) have an influence on the discriminability of stickiness intensity. Our results showed that the physical and perceived intensity of surface stickiness is different and auditory cues were the most beneficial for stickiness perception. More work will be needed to examine various aspects of surface stickiness perception, but we have provided fundamental evidence on stickiness perception by different sensory modalities. As future work, we will study which sensory modality dominates in stickiness perception using multimodal and incongruent conditions, etc.

## Data Availability

All datasets generated for this study are included in the manuscript and/or Supplementary Files.

## Ethics Statement

Experimental procedures were approved by the Ethical Committee of Sungkyunkwan University (IRB# 2018-05-001) and the study was conducted in accordance with the Declaration of Helsinki. All participants were informed about the experimental procedure and gave informed consent prior to their participation.

## Author Contributions

HL, EL, and JJ conducted the experiments, analyzed the results, and wrote the original draft of the manuscript. JK conceived of the project and supervised the project.

## Conflict of Interest Statement

The authors declare that the research was conducted in the absence of any commercial or financial relationships that could be construed as a potential conflict of interest.

## Supplementary Material

DATASET S1 Raw behavioral data for each of the 27 participants.
